# Effects of *Bifidobacterium* BL21 and *Lacticaseibacillus* LRa05 on gut microbiota in type 2 diabetes mellitus mice

**DOI:** 10.1186/s13568-023-01603-1

**Published:** 2023-09-16

**Authors:** Zhonghui Gai, Wenyan Liao, Yue Huang, Yao Dong, Huafeng Feng, Mei Han

**Affiliations:** 1 Department of Food Science, Shanghai Business School, 2271# Zhongshanxilu Road, Shanghai, 200235 China; 2 State Key Laboratory of Dairy Biotechnology, Technology Center Bright Dairy & Food Co., Ltd., Shanghai, 200436 China; 3 Department of Research and Development, Wecare Probiotics Co., Ltd., Suzhou, 215200 China

**Keywords:** T2DM, Probiotics, Gut microbiota, Spatial structure, Inflammation

## Abstract

Gut dysbiosis causes damage to the intestinal barrier and is associated with type 2 diabetes mellitus (T2DM). We tested the potential protective effects of probiotic BL21 and LRa05 on gut microbiota in type 2 diabetes mellitus mice and determined whether these effects were related to the modulation of gut microbiota.Thirty specific pathogen-free C57BL/6J mice were randomly allocated to three groups—the (CTL) control group, HFD/STZ model (T2DM) group, and HFD/STZ-probiotic intervention (PRO) group—and intragastrically administered strains BL21 and LRa05 for 11 weeks. The administration of strains BL21 and LRa05 significantly regulated blood glucose levels, accompanied by ameliorated oxidative stress in mice. The BL21/LRa05-treated mice were protected from liver, cecal, and colon damage. Microbiota analysis showed that the cecal and fecal microbiota of the mice presented significantly different spatial distributions from one another. Principal coordinate analysis results indicated that both T2DM and the BL21/LRa05 intervention had significant effects on the cecal contents and fecal microbiota structure. In terms of the fecal microbiota, an abundance of *Akkermansia* and *Anaeroplasma* was noted in the PRO group. In terms of the cecal content microbiota, enrichment of *Akkermansia*, *Desulfovibrio*, *Bifidobacterium*, *Lactobacillus*, and *Limosilactobacillus* was noted in the PRO group. The probiotics BL21 and LRa05 prevent or ameliorate T2DM by regulating the intestinal flora and reducing inflammation and oxidative stress. Our results suggest that BL21 and LRa05 colonize in the cecum. Thus, BL21/LRa05 combined with probiotics having a strong ability to colonize in the colon may achieve better therapeutic effects in T2DM. Our study illustrated the feasibility and benefits of the combined use of probiotics and implied the importance of intervening at multiple intestinal sites in T2DM mice.

**Supplementary Information:**

The online version contains supplementary material available at 10.1186/s13568-023-01603-1.

## Introduction

Type 2 diabetes mellitus (T2DM) is a complex metabolic disorder; it has become a serious global health concern and poses a huge risk to human life and health (Chaudhury et al. 2017; Zheng et al. 2018). T2DM is a disease caused by many factors, and its occurrence and development involve a variety of molecular mechanisms associated with the gut microbiota (Duan et al. 2021). Hyperglycemia may cause dysbiosis, impacting the structure and function of gut microbiota, as well as changes in intestinal-related metabolites (Wurster et al. 2021). The gut microbiota is considered a vital organ of the human body and plays crucial roles in the pathophysiology of T2DM (Salehi-Sahlabadi et al. 2021). The gut microbiota can affect the processes of intestinal permeability, inflammation, and energy metabolism, potentially affecting the development and progression of T2DM (Cox et al. 2017; Genser et al. 2018).

Probiotics are thought to play an important role in glucose homeostasis and maintaining the balance of the gut microbiota. Increasing numbers of studies on the role of probiotics in the prevention or treatment of T2DM have been published in recent years, and targeted regulation of the gut microbiota using probiotics may be an effective strategy for managing T2DM. For example, the results of animal experiments revealed that *Lactobacillus casei* CCFM419 alleviates insulin resistance and hyperglycemia in T2DM mice (Li et al. 2017). A clinical meta-analysis summarized 28 RCT studies, as a result, taking probiotics can significantly reduce patients' fasting blood glucose (FBG), glycosylated hemoglobin, and serum cholesterol levels effectively (Rittiphairoj et al. 2021). A previous study reported that supplementation with *L. rhamnosus* LRa05 modulated the gut microbiota and promoted hepatic carbohydrate metabolism in T2DM mice, thereby ameliorating obesity (Sun et al. 2020). Further studies showed that LRa05 reduced the FBG levels, insulin resistance (IR), inflammation, and hepatic oxidative stress in T2DM mice (Wu et al. 2021). In addition, we previously showed that BL21 positively affected the gut microbiota and ameliorated hepatic metabolic abnormalities in high-fat diet (HFD)-induced obese mice and streptozotocin (STZ)-induced T2DM mice and that BL21 decreased the elevated FBG and fasting serum insulin levels in T2DM mice (Wu et al. 2020b; Hao et al. 2022). Besides, both BL21 and LRa05 can reduce the relative abundance of harmful bacteria, like *Odoribacter* and *Mucispirillum*, while increasing the relative abundance of beneficial bacteria, like *Akkermansia* and *Alloprevotella* (Sun et al. 2020). All these studies suggested that probiotics can improve T2DM by modulating the gut microbiota. However, few studies have reported on the effects of compound probiotics as well as probiotics on the spatial structure of the intestine.

Because fecal sampling is an easy process, feces are often used as a proxy to study the gut microbiota. However, the mammalian gut is a complex ecosystem having multiple compartments and different physicochemical and nutritional conditions, thereby housing varying microbial communities (Stevens and Hume 1998; Hume 2002). The gut microbiota establishes a close symbiotic relationship with its mammalian host (Ley et al. 2008). Based on the varying physicochemical conditions, food pellets may be colonized by different microbiota in different areas along the longitudinal axis of the gut (Stevens and Hume 1998; Hume 2002). Bacterial communities from different intestinal sites may have different compositions and functions (Eckburg et al. 2005; Durbán et al. 2011; Stearns et al. 2011; Zoetendal et al. 2012); however, most studies use only fecal samples to represent the intestinal microbiota. To understand the effects of T2DM and probiotics on the spatial heterogeneity of the gut microbiota, we investigated the effects of strains *Bifidobacterium longum* subsp. *longum* BL21 and *L. rhamnosus* LRa05 on the cecal contents and colonic terminal (fecal) microbiota in T2DM mice.

In this study, changes in the FBG levels, immune regulation-related inflammatory factors, and oxidative stress were observed after the intragastric administration of strains BL21 and LRa05 in an HFD/STZ-induced T2DM mouse model. Streptozotocin (STZ) specifically induces DNA strand breaks in β-cells and is a common method for the establishment of diabetic models (Patel et al. 2012). To investigate the spatial heterogeneity of the gut microbiota, a culture-independent 16S rRNA gene sequencing technology was used to investigate the bacterial communities in the cecum and feces. The gut microbiota community characteristics were simultaneously characterized and compared in terms of species composition, diversity, and abundance as well as taxonomic information. The results of our study will provide valuable data for further exploring the effects of probiotics in alleviating T2DM in terms of passing through the gut microbiota.

## Material and methods

### Preparation of bacterial suspensions

Strains BL21 and LRa05 were cultured in De Man, Rogosa, and Sharpe broth at 37 °C for 18 h, and the cell pellets were collected by centrifugation (4500 g, 10 min). The pellets were suspended in sterilized water (10^10^ CFU/mL) and then stored at 4 °C until further use. The suspension was prepared daily and administered to the mice, with a dose of 1 × 10^9^ CFU being the same as the amount used in our previous work.

### Experimental animals

Thirty specific-pathogen-free male C57BL/6 J mice (six week old) were obtained from Shanghai Laboratory Animal Center, China. The mice were housed in an environment with a constant temperature (22 ± 2 °C), humidity (65% ± 5%), and a 12-h light/dark cycle. All the mice had ad libitum access to water and food. The basic diets fed to the mice per kilogram contained Casein (80 Mesh) 200 g, L-Cystine 3 g, Corn Starch 397 g, Maltodextrin 10 132 g, Sucrose100 g, Cellulose BW200 50 g, Soybean Oil 70 g, TBHQ 0.014 g, Mineral Mix S10022G 35 g, Vitamin Mix 10 g, Choline Bitartrate 2.5 g. All the experimental procedures performed in this study were approved by the Animal Care and Use Committee of Shanghai Laboratory Animal Center (No. 2022032001).

### Animal experimental design

**Fig. 1:**
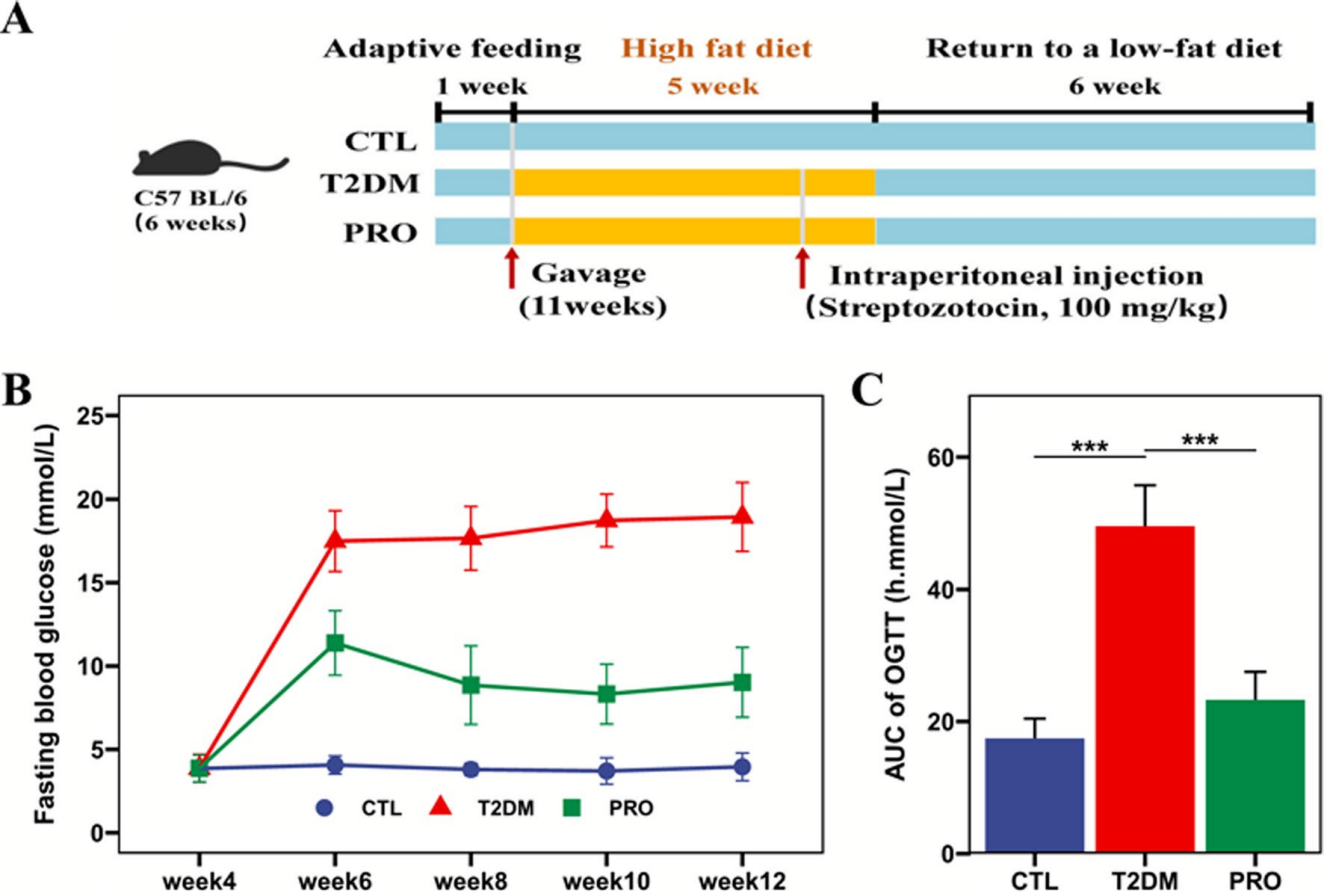
Effects of BL21 and LRa05 on glucose homeostasis in T2DM mice. Schematic diagram of test flow (**A**), fasting blood glucose level during the test (**B**), and area under the curve of oral glucose tolerance test at week 12 (**C**) in T2DM mice. Data are presented as mean and standard deviation (SD) in B and C. CTL: control; T2DM: type 2 diabetes mellitus; PRO: BL21 and LRa05 mixture (109 CFU each). *p < 0.05, **p < 0.01 and ***p < 0.001 vs T2DM group

The experimental design is presented in Fig. 1A. After 1 week of adaptive feeding, the mice were randomly divided into three groups (n = 10/group): the control (CTL) group (healthy control mice not treated with HFD/STZ but orally administered 0.2 mL of sterilized water); the T2DM group (HFD/STZ-induced mice; orally administered 0.2 mL of sterilized water by gavage); and the probiotic BL21/LRa05 intervention (PRO) group. From week 1 to 12, the CTL group was fed a standard chow diet, whereas the other two groups were fed an HFD for 5 weeks. The PRO group was also administered strains BL21 and LRa05 (10^9^ CFU/each mouse) at weeks 2–12. On day 1 of week 5, the T2DM and PRO groups were fasted for 12 h and injected with freshly prepared STZ (50 mmol/L; 100 mg/kg body weight), whereas the CTL group was injected with 0.85% saline. The FBG concentrations were measured at 1 week after the STZ injection, and the FBG was found to be > 7.0 mmol/L in the T2DM mice after successful modeling (Wu et al. 2021; Hao et al. 2022).

### Determination of the FBG levels and oral glucose tolerance in the mice

Blood samples were collected from the caudal vein and centrifuged at 4,000 g (4 °C for 10 min) for serum separation. The blood glucose levels were measured using Accu-Check Performa Blood Glucose Test Strips (Roche Applied Science). The FBG level was measured every 2 weeks after 12 h of water fasting. An oral glucose tolerance test was performed after successful modeling and at the end of the study. The mice were fasted for 12 h, and their FBG levels were measured (0 min); they were then intragastrically administered a 2 g/kg glucose solution, and their blood glucose levels were again measured at 30, 60, 90, and 120 min. A curve was constructed using the determined blood glucose values, and the area under the curve (AUC) was calculated.

### Collection of blood, tissue samples, mouse feces, and colonic content samples

The mice were placed in a heat-sterilized cage and allowed to defecate naturally. Then, their feces were collected and stored at − 80 °C for microbiota analysis. At the end of the study (week 12), the mice were fasted for 12 h and anesthetized with isoflurane. Blood samples were collected from the inferior vena cava, centrifuged at 4000 × *g* for 10 min at 4 °C to separate the serum, and stored at − 80 °C until further use. The liver, cecal, and colon tissue samples were then extracted rapidly and stored at − 80 °C or fixed in 4% formaldehyde solution. Moreover, the colonic contents were collected and immediately stored at − 80 °C for subsequent microbiota analysis.

### Histological analysis

Optimal cutting temperature compound-embedded liver tissues were cut into 4-μm-thick sections and then incubated with 5 μmol/L dihydroethidium (DHE) for 30 min at 37 °C under dark conditions to observe ethidium fluorescence. DHE was also applied to detect the hepatic reactive oxygen species (ROS) levels. The formaldehyde-fixed liver and cecal tissues were sectioned after embedding in paraffin, cut into 5-µm-thick sections, and stained with hematoxylin and eosin. Moreover, the liver tissue samples were subjected to Masson’s trichrome staining for observing the collagen fiber status.

### Detection of inflammation

The levels of serum lipopolysaccharide (LPS), tumor necrosis factor alpha (TNFα), interleukin (IL)-1β, IL-6, and IL-10 were measured using enzyme-linked immunosorbent assay kits (Wuhan Chundu Biotechnology Co., Ltd., China) by microplate reader (ReadMax 1900Plus, Flash Spectrum Biological Technology, Shanghai, China), according to the manufacturer’s instructions.

### DNA extraction and pyrosequencing

DNA extraction and pyrosequencing were conducted as described in our previous study (Dong et al. 2022). Briefly, DNA was extracted, after which the V3–V4 region of the 16S rRNA gene was amplified using 341F and 805R primers and sequenced using the MiSeq platform (Illumina) using a 2 × 300-bp paired-end read.

### Bioinformatics analysis of the 16S rRNA gene amplicons

The bioinformatics analysis results of the sequenced amplicons are presented in our previous manuscripts (Dong et al. 2022; Han et al. 2022). The sequences were analyzed using the Usearch v11 sequence analysis tool (Edgar 2016) and annotated using the 16S rRNA reference training set of the Ribosomal Database Project (v18) (https://www.drive5.com/usearch/manual). Based on an amplicon sequence variant table, an alpha diversity analysis was performed using the alpha.div function of the Usearch v11 tool.

### Statistical analysis

Student's t-tests were used for continuous variables that conformed to a normal distribution, such as fasting blood glucose and area under the curve of oral glucose tolerance. The nonparametric Kruskal–Wallis test was used for variables that did not conform to a normal distribution. Significant between-group differences were analyzed using the nonparametric Mann–Whitney *U*-test. Linear discriminant analysis combined with effect size (LEfSe) measurement was used to identify biomarkers unique to each group based on the abundance values (Langille et al. 2013). Principal coordinate analysis (PCoA) of the microbiota data ordination plots was based on Bray distances for beta diversity analysis, and the significant between-group differences were determined using the adonis2 function in the R vegan package (Oksanen et al. 2019). The Mantel test function in the R vegan package was used to test the correlations among the cecal contents and fecal microbiota based on the Bray distances. The microbiome statistics were assessed, and a graph was generated using the ggplot2 software package (Gómez-Rubio 2017). All statistical analyses were performed using R v4.2 (Team 2013), and p < 0.05 was considered statistically significant.

## Results

### Effects of probiotics on the FBG levels and glucose tolerance in T2DM mice

Two weeks after the STZ injection, the mice in the CTL group exhibited a bright coat color, were energetic, and fed on a normal diet and water. However, the T2DM and PRO groups lacked energy at varying degrees, exhibited a dim coat color, and showed markedly increased drinking water consumption and urine output. Figure 1B shows the FBG levels for each group. After the STZ injection, the FBG levels were significantly higher in the T2DM group than in the CTL group and remained high until the end of the experiment. By contrast, the glucose levels were significantly lower in the PRO group than in the T2DM group. As shown in Fig. 1C, based on the oral glucose tolerance test results, glucose tolerance was severely impaired in the T2DM group; moreover, the glucose AUC of the graphs plotted using these results was significantly greater for the T2DM group than for the CTL group. Compared with that in the T2DM group, the glucose AUC was significantly lower after the administration of probiotic BL21/LRa05 by oral gavage. These results indicated that BL21/LRa05 significantly improved the FBG level and glucose tolerance in T2DM mice.

### Protective effect of probiotics on the liver of T2DM mice

**Fig. 2:**
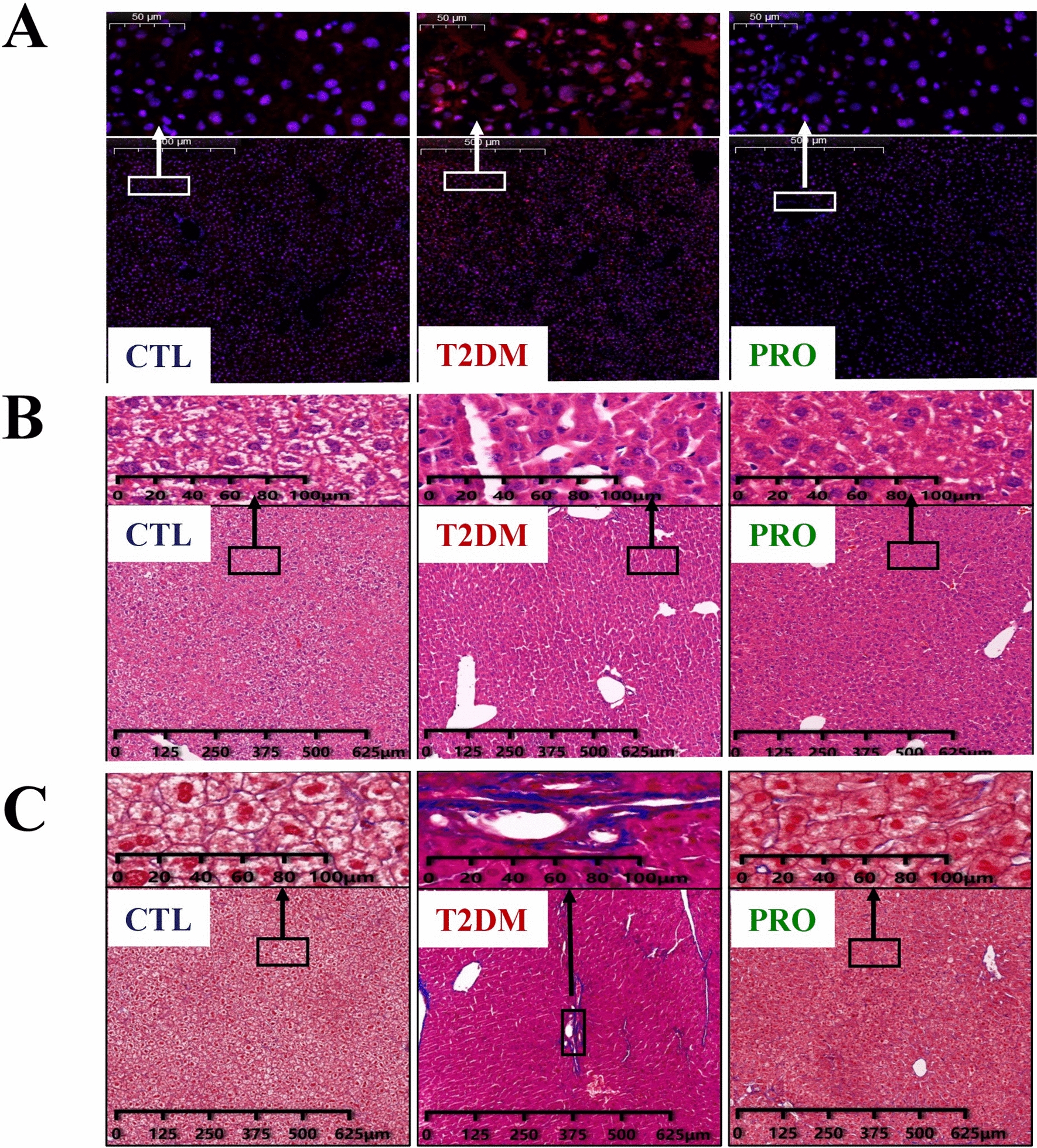
Representative optical micrographs of livers stained with DHE (**A**), HE (**B**), and Masson (**C**)

Increased DHE oxidation was observed in the T2DM group compared with the CTL group (Fig. 2A), implying increased hepatic ROS levels in the T2DM group. Compared with the T2DM group, the PRO group demonstrated ameliorated hepatic ROS abnormalities.

The liver tissue hematoxylin and eosin staining results are shown in Fig. 2B. The hepatic lobule was intact and clear and there was almost no lipid deposition in the cells in the CTL group. Necrosis in the lobules and inflammatory cells in the portal area were observed in the hepatocytes in the T2DM group. The BL21/LRa05-supplemented diet significantly improved the pathological appearance of these structures.

The liver tissue Masson’s trichome staining results are shown in Fig. 2C. The hepatic cords in the CTL group were clear and neatly arranged, and a small amount of collagen fibers, which were blue, were observed in the portal area. Collagen fiber staining was significantly increased in the T2DM group compared with the CTL group, and most of the fibers were distributed in the portal and perivascular areas. The collagen fiber tissue was significantly reduced in the PRO group compared with the T2DM group.

### Effect of probiotics on serum inflammatory factors in T2DM mice

**Fig. 3:**
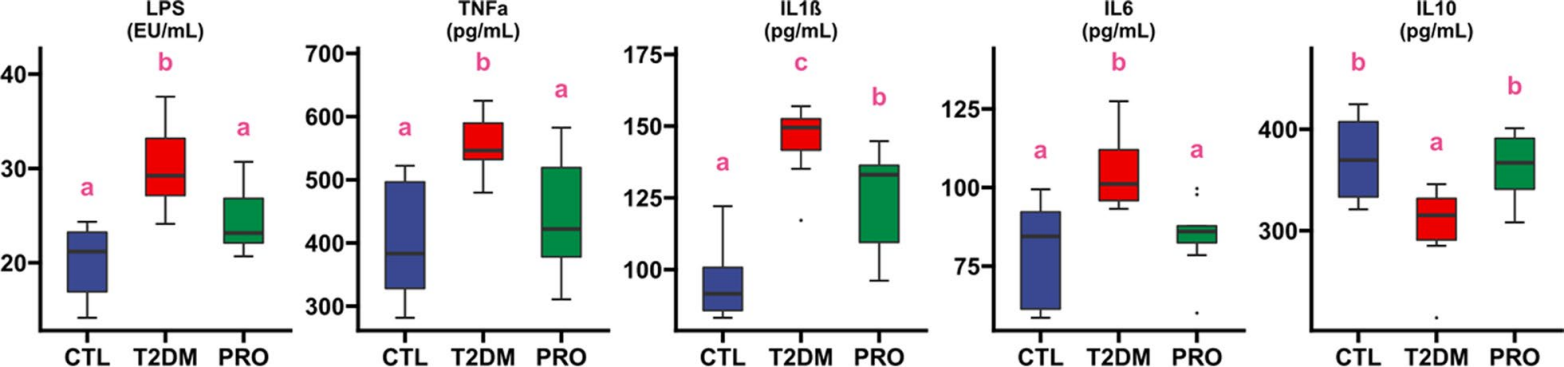
Effect of BL21/LRa05 on serum LPS and inflammatory cytokines in T2DM mice. Different letters indicate significant differences between groups (p < 0.05), box plot represents interquartile range, the line within the box represents median value

Bacterial LPS can cause inflammation (Gong et al. 2018). BL21/LRa05 significantly reduced inflammation in the T2DM mice. As shown in Fig. 3, the levels of serum LPS, TNFα, IL1β, and IL6 were significantly higher in the T2DM group than in the CTL group. The BL21/LRa05 intervention significantly decreased the levels of serum LPS and inflammatory factors, particularly TNFα and IL6, compared with those in the T2DM mice, the levels in which were not significantly different from those in the CTL group. The IL10 levels were significantly lower in the T2DM mice than in the normal mice. Moreover, the BL21/LRa05 intervention effectively improved the serum IL-10 levels. The above results show that the BL21/LRa05 intervention could effectively reduce the proinflammatory factor levels and increase the anti-inflammatory factor levels, indicating that BL21/LRa05 could efficiently regulate the inflammatory factor levels in T2DM mice.

### Effects of T2DM and probiotics on the fecal and cecal content microbiota

**Fig. 4:**
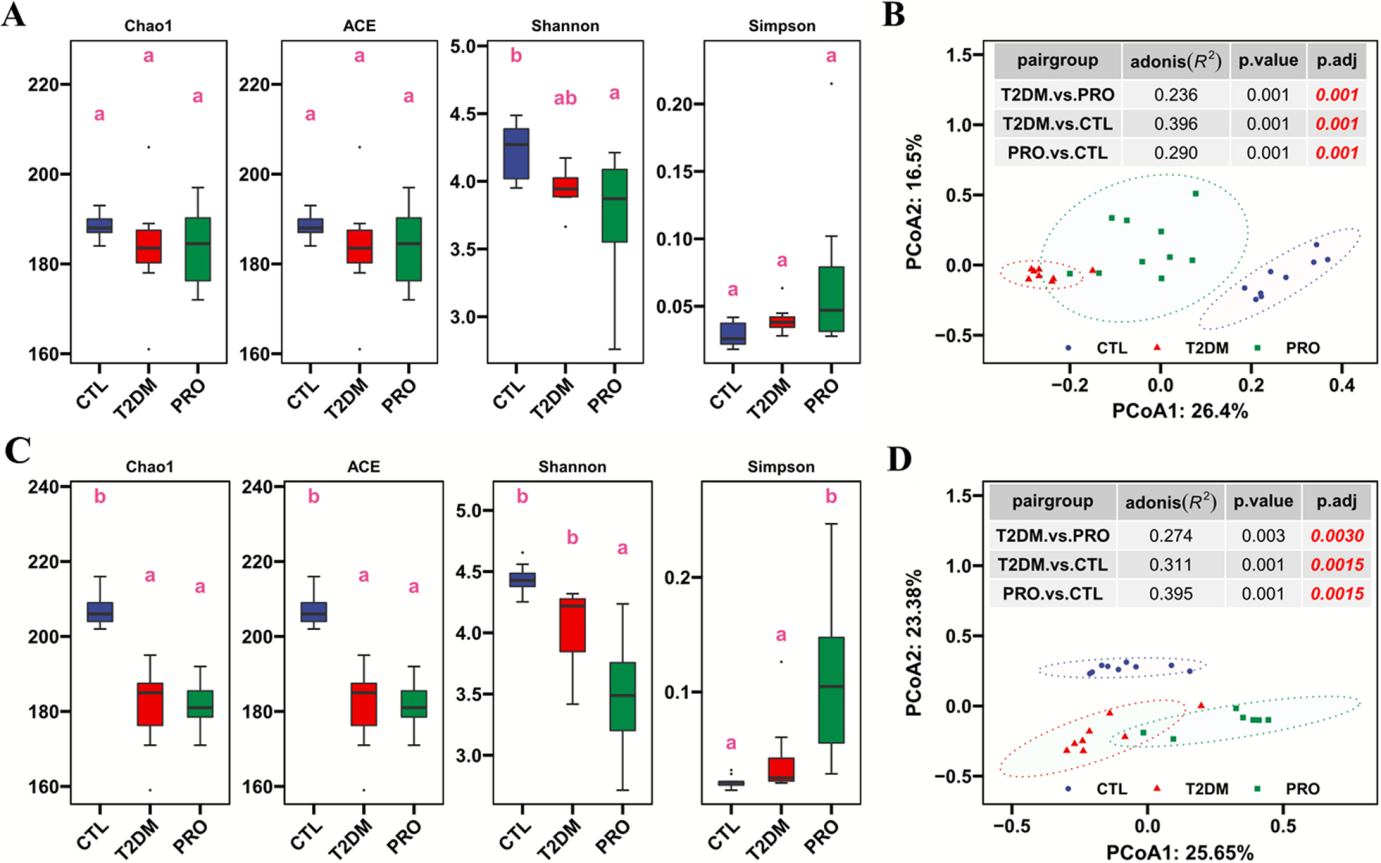
Effects of probiotics on diversity of fecal and cecal content microbiota in T2DM mice. Effect of probiotics on alpha diversity (**A**) and beta diversity (**B**) of fecal microbiota in T2DM mice. Effect of probiotics on alpha diversity (**C**) and beta diversity (**D**) of cecal content microbiota. Alpha diversity (**A** and **C**) was represented by boxplots, and significant differences were represented by lowercase letters, with different letters indicating significant differences between the two groups. PCoA analysis was used to demonstrate beta diversity (**B** and **D**) between groups, and adonis analysis was used to evaluate the significance of differences between groups (tables in **B** and **E**). Box plot represents interquartile range, the line within the box represents median value

As shown in Fig. 4A, there was no significant difference in the abundance of the fecal microbiota among the three groups (as determined using the Chao1 estimator and ACE index); however, the diversity was significantly reduced in the PRO group (based on the Shannon index). The PCoA results revealed significant separation among the three groups, indicating that the BL21/LRa05 intervention had a significant effect on the fecal microbiota structure (Fig. 4B). Unlike the fecal microbiota, the cecal contents in the T2DM and PRO groups exhibited a significantly lower abundance of the fecal microbiota (as determined using the Chao1 estimator and ACE index) compared with that in the CTL group (Fig. 4C). Moreover, the diversity of the cecal microbiota was significantly reduced in the PRO group (as determined using the Shannon and Simpson indexes). The PCoA results also showed significant differences in the cecal content microbiota among the three groups (Fig. 4D).

A beta diversity analysis was performed, which showed significant differences in the fecal and cecal content microbiota among the three groups; accordingly, we further analyzed the composition of the microbiota at the phylum level. As shown in Additional file 1: Fig. S1, *Bacteroidetes*, *Firmicutes*, *Proteobacteria*, *Candidatus*_*Saccharibacteria*, *Actinobacteria*, and *Verrucomicrobia* were the major constituents of the fecal and cecal content microbiota, and their relative abundance accounted for more than 99% of the entire flora. Of all these, *Bacteroidetes* and *Firmicutes* were the most abundant, accounting for more than 80%; however, the relative abundance of *Firmicutes* in the cecal contents was higher than that of *Bacteroidetes*.

**Fig. 5:**
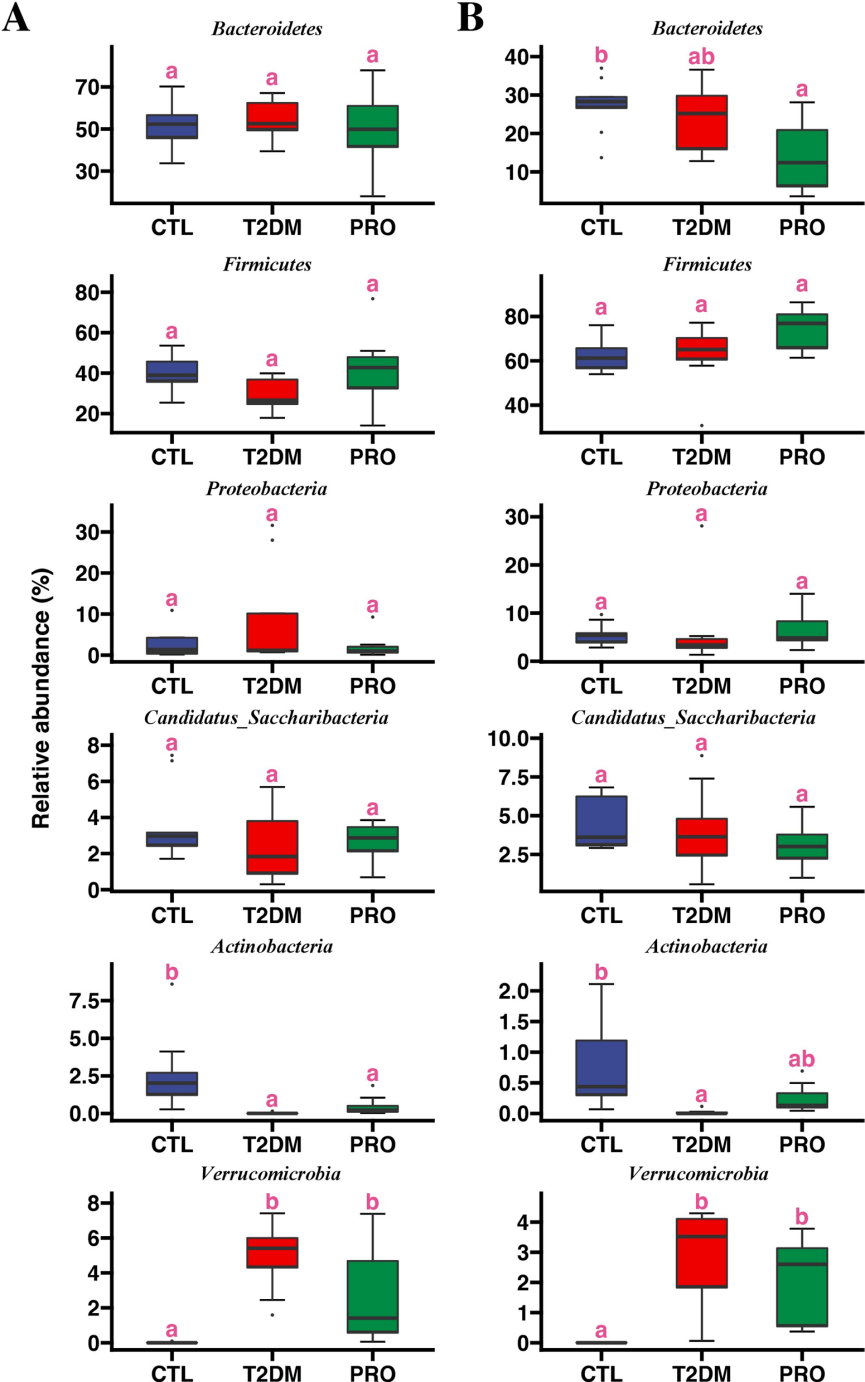
Composition analysis of microbiota structures at phylum level for fecal (**A**) and cecal contents (**B**). Different letters indicate significant differences between groups (p < 0.05). Box plot represents interquartile range, the line within the box represents median value

In addition, significant spatial heterogeneity in terms of the bacterial species composition was found at certain intestinal sites (Additional file 1: Fig. S1). Except for a significant decrease in the abundance of *Bacteroidetes* in the PRO group, statistical analyses revealed no significant differences in the abundance of *Bacteroidetes*, *Firmicutes*, *Proteobacteria*, and *Candidatus*_*Saccharibacteria* in the fecal and cecal contents (Fig. 5). Compared with that in the CTL group, the abundance of *Actinobacteria* was significantly lower in the T2DM group and tended to be higher in the PRO group, particularly in the cecal region (Fig. 5). In contrast, the abundance of *Verrucomicrobia* was significantly increased in the T2DM group but tended to decrease in both the fecal and colonic contents after the BL21/LRa05 intervention.

### Specific bacteria at different sites

**Fig. 6:**
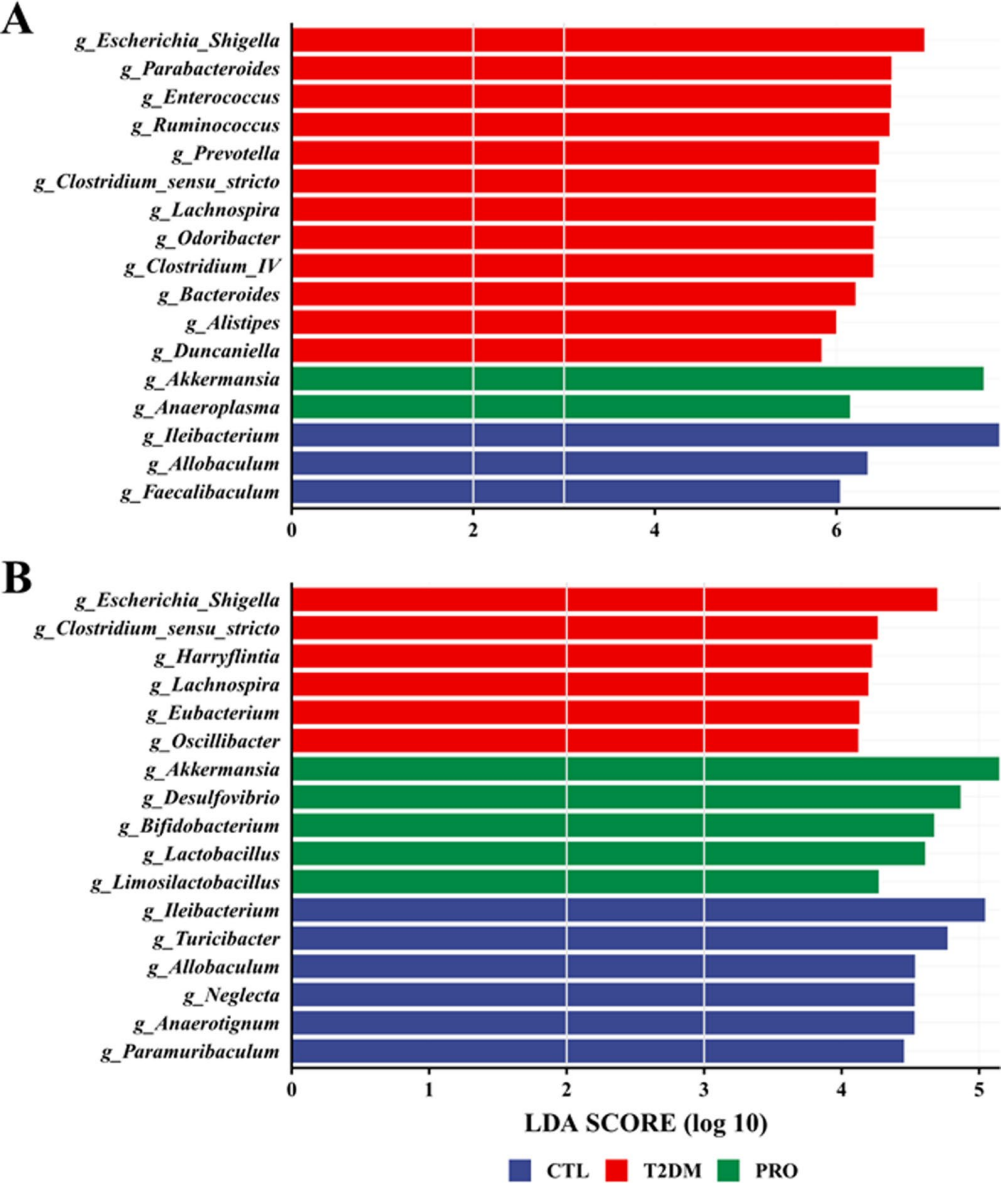
Results of Linear discriminant analysis effect size (LEfSe) analysis at genus level. T2DM caused an increases of *Escherichia/Shigella, Parabacteroides, Enterococcus* in faces samples (**A**). Compared with the T2DM group, supplementation with BL21/LRa05 increased the relative abundance of some beneficial bacteria, such as *Akkermansia*, *Desulfovibrio, Bifidobacterium, Lactobacillus*, and *Limosilactobacillus* (**B**). LEfSe analysis calculates the linear discriminant analysis (LDA) score by LDA effect size, which is assessed for each differentially abundant bacterial taxon using LDA

Because both habitat conditions and disease can significantly affect the gut bacteria, we further analyzed the differences in the fecal and cecal content microbiota among the three groups using LEfSe analysis (Fig. 6); the results revealed that in terms of the fecal microbiome, the T2DM group was mainly enriched with *Escherichia/Shigella*, *Parabacteroides*, *Enterococcus*, *Ruminococcus*, *Lachnospira*, and *Odoribacter*, whereas the PRO group was abundant in *Akkermansia* and *Anaeroplasma*. In terms of the cecal microbiome, the T2DM group was found to be abundant in *Escherichia*/*Shigella* and *Lachnospira*. Notably, we found that the PRO group was enriched in *Akkermansia*, *Desulfovibrio*, *Bifidobacterium*, *Lactobacillus*, and *Limosilactobacillus*.

The Mantel test helps determine the correlation between two sets of distance measure matrixes, rather than two sets of variable matrixes, and is used to determine whether the sample distances in one matrix correlate with those in the other matrix. The Mantel test revealed a correlation between the cecal contents and fecal microbiota in the CTL and T2DM groups, and the correlation coefficient was found to be greater in the CTL group (Additional file 1: Fig. S2A and B). However, no association was found between the cecal and fecal content microbiota in the PRO group. This corroborated the results of the LEfSe analysis, implying that BL21/LRa05 colonize the cecum and thus have a greater impact on the cecal content microbiota structure.

### Correlation analysis of glycemic status and gut microbiota

Additionally, as shown in Additional file 1: Fig. S3, a correlation analysis between glycemic status and gut microbiota was performed. It can be observed that *Escherichia/Shigella*, *Clostridium_sensu_stricto*, *Lachnospira*, *Parabacteroides*, and *Enterococcus* enriched in the T2DM group were positively correlated with FBG and OGTT. Whereas some bacteria enriched in the PRO group such as *Akkermansia*, *Bifidobacterium*, *Desulfovibrio*, and *Lactobacillus* were negatively correlated with FBG and OGTT. Also, bacteria enriched in CTL groups such as *Paramuribaculum* and *Faecalibaculum* were negatively correlated with FBG and OGTT. These results implied the association of disturbed gut microbiota with T2DM and the beneficial effect of PRO on gut microbiota improvement.

### Histopathology of the cecum and colon

**Fig. 7:**
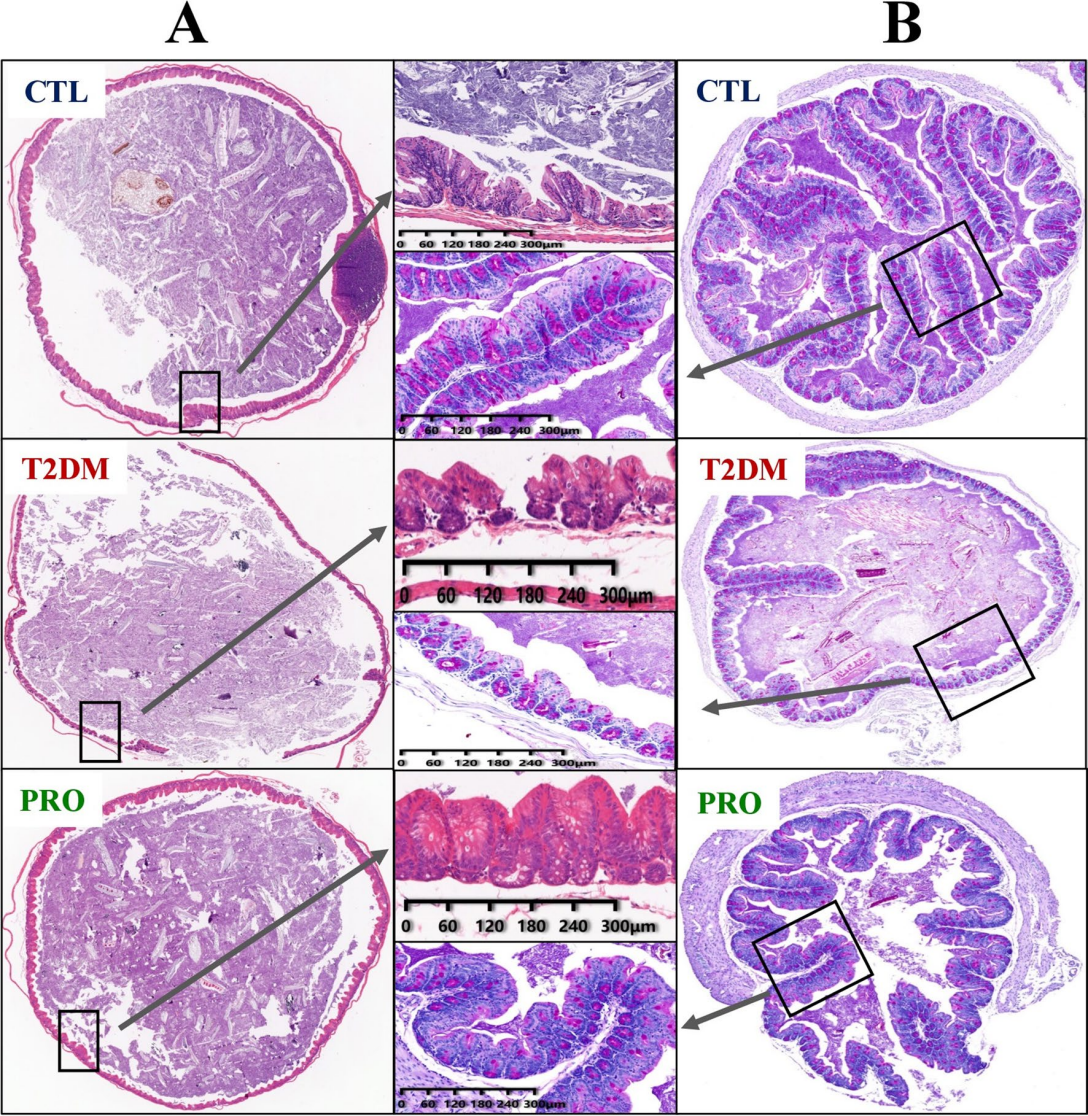
Representative optical micrographs of cecal and colonic tissues stained with H&E (**A**) and periodic acid-Schiff (**B**)

The results of tissue staining revealed that the BL21/LRa05 intervention ameliorated the damage caused by gut dysbiosis to the cecum and colon. As shown in Fig. 7, the cecal glandular structures in the CTL group were neatly arranged in the lamina propria, without any breakage. In the T2DM group, the intestinal gland structure was disordered, and there were a large number of breaks. Edema was observed to an extent; moreover, the thickness of the muscularis mucosae was inconsistent between groups, with that in the T2DM group becoming significantly thinner. The BL21/LRa05 intervention significantly improved the cecal tissue structure as compared with that in the T2DM group. These results indicated that the BL21/LRa05 intervention could protect the cecal tissue structure in T2DM mice. In addition, according to the periodic acid–Schiff staining results (Fig. 7B), the mucus layer of the colon in the T2DM group showed significant destruction, the mucin content was significantly reduced, and the goblet cells had almost disappeared. By contrast, the BL21/LRa05 intervention significantly increased the number of goblet cells, improved the distribution of mucin, and protected against damage to the colonic mucus layer in T2DM mice.

## Discussion

Herein, we revealed the antidiabetic effects of the probiotics BL21/LRa05 on HFD/STZ-induced T2DM mice. BL21/LRa05 administration significantly improved hyperglycemia and gut dysbiosis. Interestingly, most of the biochemical outcomes achieved by the composite probiotic BL21/LRa05 intervention were comparable to those observed in healthy mice, suggesting that the intervention has beneficial effects in preventing or improving T2DM.

Oxidative stress plays an important role in IR and T2DM development (Houstis et al. 2006). T2DM is characterized by hyperglycemia and dyslipidemia, which can trigger an increase in ROS generation, thereby leading to organ damage (e.g., liver damage). Some probiotics have antioxidant functions (Amaretti et al. 2013). *L. rhamnosus*, *L. helveticus*, and *L. casei* reportedly decreased the malondialdehyde levels and increased the superoxide dismutase (SOD) and catalase levels in cadmium-treated rats (Dashtbanei and Keshtmand 2022). Our previous study also showed that LRa05 significantly increased the catalase and glutathione activities and decreased the malondialdehyde levels in mice (Wu et al. 2021). The increase in the SOD content in the PRO group was associated with BL21, based on our recent study that demonstrated that BL21 can significantly increase the SOD content in the liver of T2DM mice (Hao et al. 2022). These results suggested that the combination of BL21 and LRa05 can better protect the T2DM mice from oxidative damage, thereby improving IR and reducing tissue lesions. This protective effect was also confirmed by performing histological hepatic staining (Fig. 2). In particular, compared with that in the T2DM group, the BL21/LRa05 intervention reduced the hepatic collagen fiber content, thereby relieving fibrosis in the liver.

Studies have shown that gut microbiota composition is disturbed to varying degrees in T2DM patients compared to healthy controls. The causal relationship between T2DM and disturbed gut microbiota is currently unknown and requires further investigation. Increasing numbers of studies have linked gut dysbiosis to T2DM development. A disturbed gut microbiota associated with T2DM leads to increased gut permeability; moreover, some proinflammatory substances such as LPS enter the liver and induce low-grade inflammation, in turn triggering IR (Vijay-Kumar et al. 2010). Compared with single bacteria, multiple bacteria may play more effective roles in regulating the gut microbiota. Multiple strains of probiotics play beneficial roles in inducing IR through the probiotic–gut microbiota–butyrate inflammatory pathway (Li et al. 2016). Similar findings have been reported in other areas, e.g., in a meta-analysis of the effects of probiotics on preterm infants, and the results showed that single probiotic supplements had limited efficacy compared with combinations of probiotics; moreover, a combination of *Lactobacillus* and *Bifidobacterium* was recommended to achieve the best effects on the health of preterm infants (Chi et al. 2021). The combination of BL21 and LRa05 significantly modulated the gut microbiota and inflammatory responses. In particular, this combination not only decreased the serum LPS and proinflammatory factor levels but also significantly increased the IL10 levels (Fig. 3), a finding that was not observed when we previously intervened using single bacteria. Furthermore, T2DM is associated with reduced abundance of a SCFAs-producing organisms, particularly those producing butyrate, which is associated with insulin sensitivity (Lappi et al. 2014). In this study, correlation analysis showed that *Akkermansia*, *Bifidobacterium*, *Desulfovibrio* and *Lactobacillus* were negatively correlated with FBG and OGTT, and that these microorganisms were enriched in the PRO group. The enrichment of these microorganisms caused by PRO intervention may benefit the production of SCFAs, thus playing a role in regulating gut microbiota structure, reducing the level of inflammatory factors and regulating hepatic glucose metabolism. These results suggested that the combination of BL21/LRa05 effectively alleviates IR and that this effect is related to the mechanisms regulating the gut microbiota and improving the inflammatory status.

The gut microbiota has been implicated in human health and disease; however, the feces do not completely reflect the gut microbial ecology (Lavelle et al. 2013). Our findings reveal that significant spatial heterogeneity exists in the bacterial communities of the mouse gut ecosystem and that T2DM and probiotic interventions have significant effects on the fecal and cecal content microbiota. Although significant differences were noted in the fecal and cecal content microbiota, *Escherichia/Shigella* were found to be enriched in both the fecal and cecal contents of the T2DM group. *Escherichia/Shigella* belong to the phylum *Proteobacteria* and are relatively enriched gram-negative bacteria found in patients with T2DM (Hoang et al. 2021); moreover, the increased abundance of *Proteobacteria* can lead to increased LPS levels and trigger systemic inflammatory responses (Jeong et al. 2019). *Akkermansia* is a bacterial genus that can produce propionate and butyrate, thereby inducing anti-inflammatory effects (Wu et al. 2020a). Interestingly, *Akkermansia* was enriched upon treatment with BL21/LRa05 in both the fecal and cecal content microbiota (Fig. 6). In addition, enrichment of *Bifidobacterium*, *Lactobacillus*, and *Limosilactobacillus* was noted in the cecal contents but not in the feces. These results implied that BL21 and LRa05 colonize in the cecum. The gut microbiota structure is influenced by both the local habitat conditions and individual host differences; the local habitat conditions determine the structure of the predominant community, whereas the individual host factors determine the detailed composition of the members of the concerned species (Lu et al. 2014). Although histological sections showed that BL21/LRa05 protected both the colon and cecal tissues in the mice (Fig. 7), it may be wise to select a probiotic capable of colonizing the colon in combination with BL21 and LRa05 to obtain better therapeutic outcomes.

**Fig. 8:**
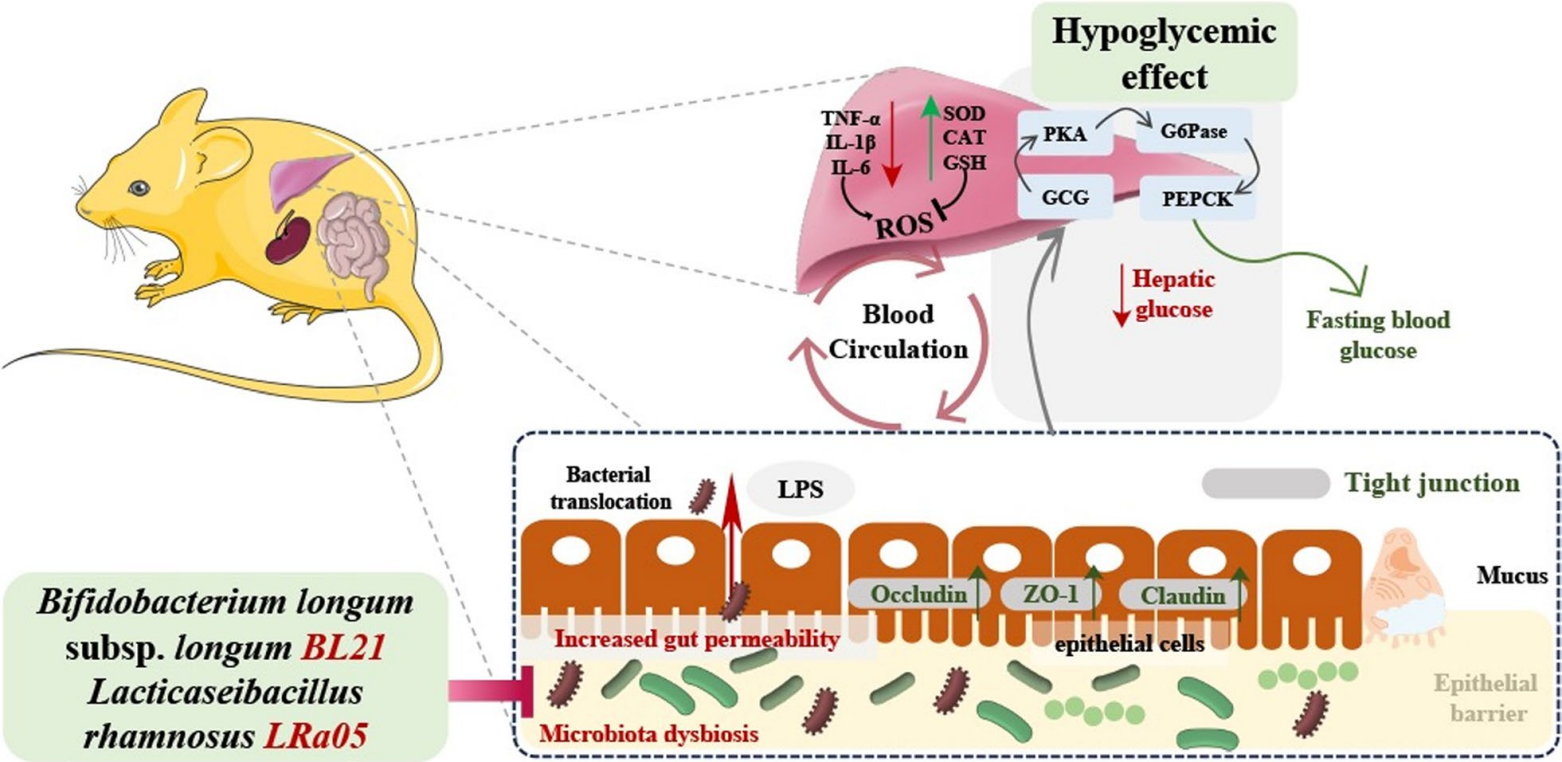
BL21/LRa05 modulates the gut microbiota to improve the pattern of type 2 diabetes mellitus (T2DM) presumably. Lipopolysaccharide (LPS), Zonula occludens-1 (ZO-1), Tumor Necrosis Factor-alpha (TNF-$\alpha$), Interleukin-1 $\upbeta$ (IL-1 $\upbeta$), (IL-6), Superoxide Dismutase (SOD), Catalase (CAT), Glutathione (GSH), Protein Kinase A (PKA), Glucose-6-phosphatase (G6Pase), Glucagon (GCG), Phosphoenolpyruvate Carboxykinase (PEPCK)

To the best of our knowledge, in this study, for the first time we investigated the effects of probiotics on the spatial structure of the gut microbiota in T2DM mice using a pair of probiotic strains. The effects of BL21 and LRa05 on FBG control and the potential underlying mechanisms were further revealed from the perspective of the gut microbiota (Fig. 8). T2DM in mice is associated with dysbiosis of the gut microbiota and heightened intestinal permeability, allowing harmful bacteria and their metabolites (e.g., LPS) to translocate into the liver through the bloodstream. This process initiates inflammatory responses and oxidative stress, influencing metabolic pathways related to glucose metabolism, leading to hepatic lipid accumulation, insulin resistance, and elevated fasting blood glucose levels. BL21/LRa05 intervention positively influences the composition and function of the gut microbiota by promoting the growth of beneficial bacteria and enhancing the production of short-chain fatty acids. This leads to improved gut barrier function and decreased intestinal permeability, consequently reducing the translocation of harmful bacteria and their metabolites to the liver. Consequently, liver inflammation and oxidative stress are attenuated. Moreover, BL21/LRa05 treatment regulates glucose metabolism by downregulating the expression of genes involved in hepatic glucose synthesis, resulting in reduced hepatic glucose production and improved fasting blood glucose levels. Additionally, BL21/LRa05 enhances insulin sensitivity, contributing to the overall amelioration of T2DM. These findings highlight the multifaceted approach of BL21/LRa05, involving gut microbiota modulation, attenuation of liver inflammation and oxidative stress, regulation of glucose metabolism, and enhancement of insulin sensitivity. Our study illustrated the feasibility and benefits of the combined use of probiotics and implied the importance of intervening at multiple intestinal sites in T2DM mice. However, a limitation of our study is that we did not investigate the microbiota in the small intestine and could therefore not evaluate the effects of probiotics on the intestinal microbiota in T2DM mice from a broader perspective.

In conclusion, we found that BL21/LRa05 produces antidiabetic effects in HFD/STZ-induced T2DM mice. We investigated the effects of T2DM on the spatial structure of colonic bacteria from the perspective of the gut microbiota. T2DM significantly altered the gut microbiota profiles, particularly the cecal contents, in the mice. Our results support the use of the BL21/LRa05 intervention for T2DM prevention. The results of this study showed that the combined use of probiotics significantly improved cecal and colonic microbiota in mice, which provided valuable data for further development of microbial compositions that could improve the regulation of the entire gut microbiota. However, further studies are needed to verify the efficacy of the BL21/LRa05 intervention through human clinical trials.

### Supplementary Information

**Additional file 1: Figure S1.** Distribution of gut microbiota abundance at the phylum level across different groups for fecal (**A**) and cecal contents (**B**). **Figure S2.** Results of Mantel tests to investigate correlations between the Bray–Curtis distance matrices of the gut microbiota for fecal and cecal contents in CTL (**A**), T2DM (**B**), and PRO (**C**) group. **Figure S3.** Correlation analysis of glycemic status and gut microbiota for fecal (**A**) and cecal contents (**B**).

## Data Availability

The datasets presented in this study can be found in online repositories. The names of the accession number can be found below: https://www.ncbi.nlm.nih.gov/, PRJNA942453.
