# Supplementary material for: Effects of Bifidobacterium BL21 and Lacticaseibacillus LRa05 on gut microbiota in type 2 diabetes mellitus mice
Source: AMB Express. 2023 Sep 16;13:97. doi: 10.1186/s13568-023-01603-1 (PMC10505128; doi:10.1186/s13568-023-01603-1)

**Figure S1.** Distribution of gut microbiota abundance at the phylum level across different groups for fecal (A) and cecal contents (B).


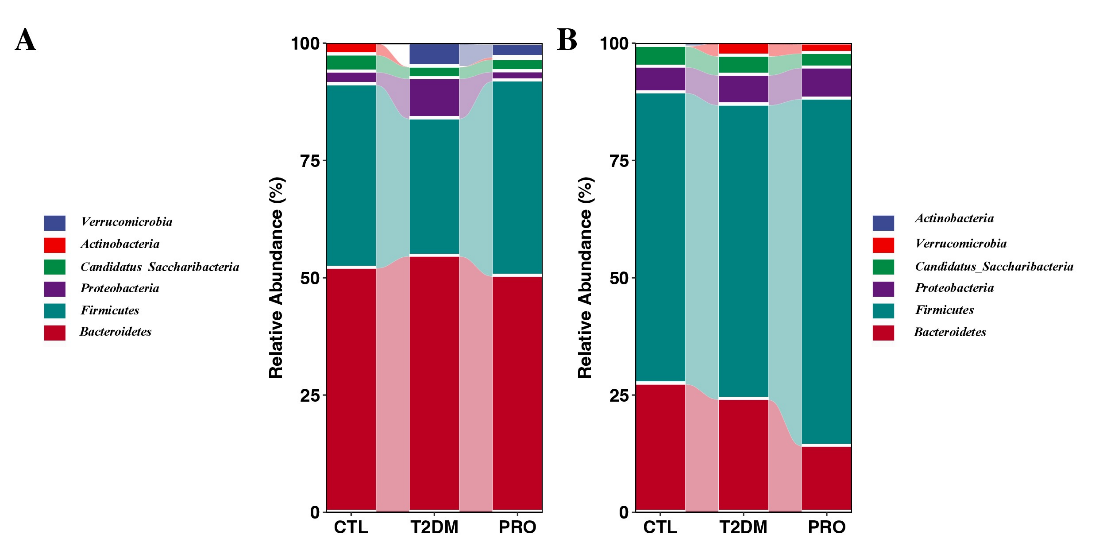


**Figure S2.** Results of Mantel tests to investigate correlations between the Bray–Curtis distance matrices of the gut microbiota for fecal and cecal contents in CTL (A), T2DM (B), and PRO (C) group.


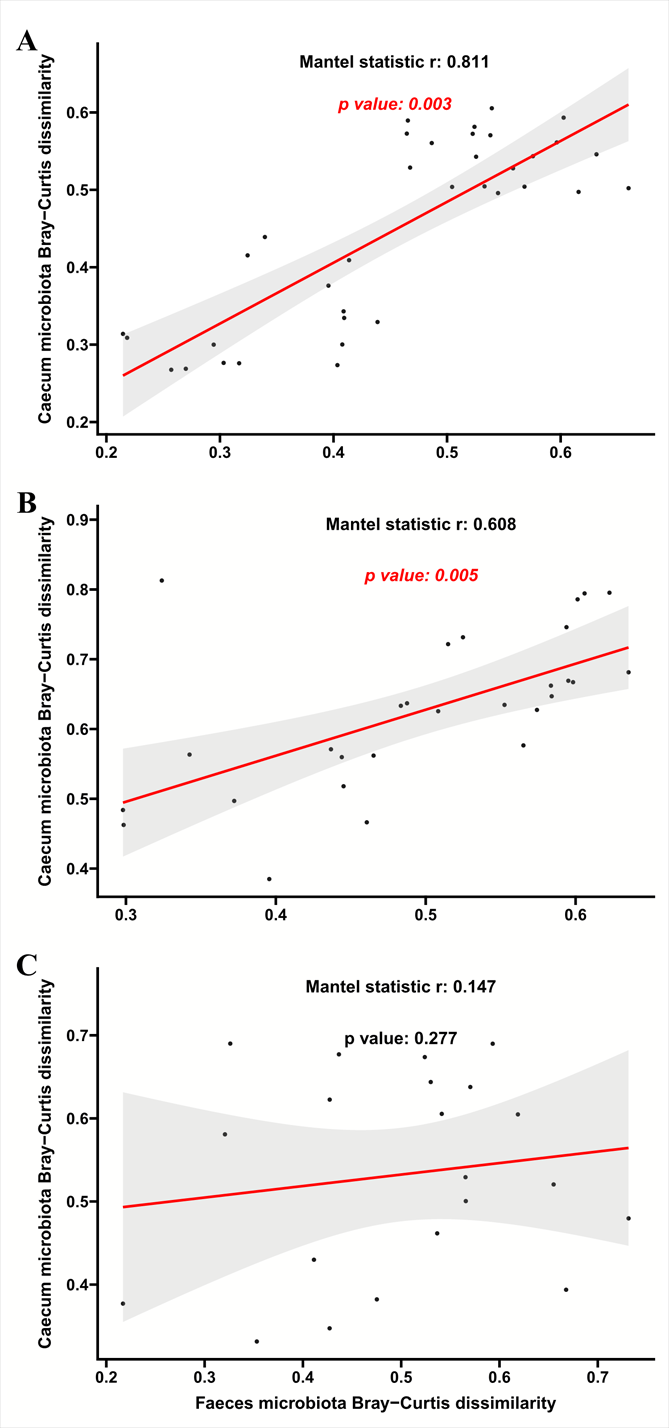


**Figure S3.** Correlation analysis of glycemic status and gut microbiota for fecal (A) and cecal contents (B).


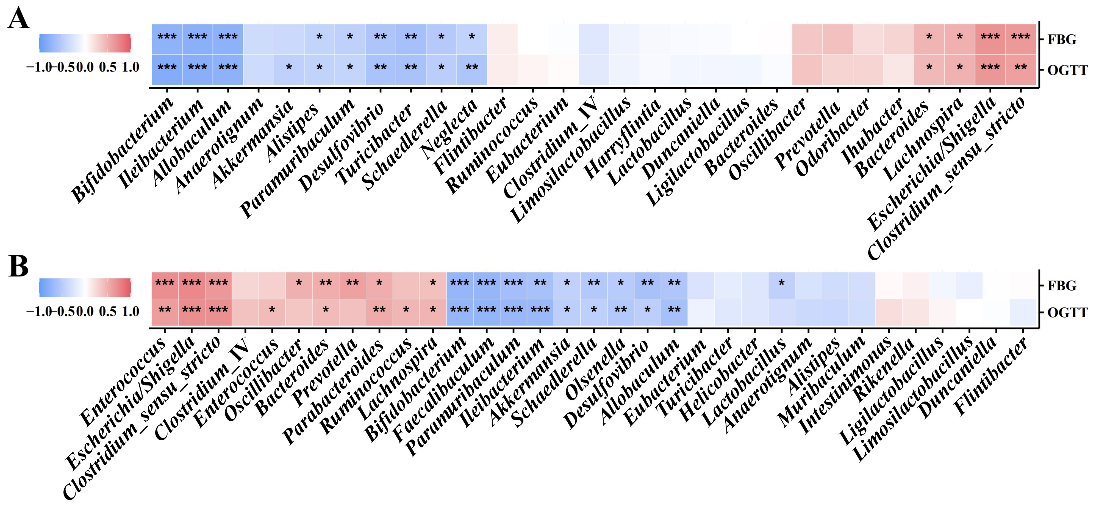

Supplement: Supplementary file 1 — Additional file 1: Figure S1. Distribution of gut microbiota abundance at the phylum level across different groups for fecal (A) and cecal contents (B). Figure S2. Results of Mantel tests to investigate correlations between the Bray–Curtis distance matrices of the gut microbiota for fecal and cecal contents in CTL (A), T2DM (B), and PRO (C) group. Figure S3. Correlation analysis of glycemic status and gut microbiota for fecal (A) and cecal contents (B). [file 13568_2023_1603_MOESM1_ESM.docx]
